# The Balance between Protealysin and Its Substrate, the Outer Membrane Protein OmpX, Regulates *Serratia proteamaculans* Invasion

**DOI:** 10.3390/ijms25116159

**Published:** 2024-06-03

**Authors:** Olga Tsaplina

**Affiliations:** Institute of Cytology, Russian Academy of Sciences, Tikhoretsky av. 4, 194064 St. Petersburg, Russia; tsaplina@incras.ru; Tel.: +7-812-297-42-96

**Keywords:** bacterial invasion, protealysin, outer membrane protein OmpX, *Serratia proteamaculans*, EGFR, β1 integrin, fibronectin, E-cadherin

## Abstract

*Serratia* are opportunistic bacteria, causing infections in plants, insects, animals and humans under certain conditions. The development of bacterial infection in the human body involves several stages of host–pathogen interaction, including entry into non-phagocytic cells to evade host immune cells. The facultative pathogen *Serratia proteamaculans* is capable of penetrating eukaryotic cells. These bacteria synthesize an actin-specific metalloprotease named protealysin. After transformation with a plasmid carrying the protealysin gene, noninvasive *E. coli* penetrate eukaryotic cells. This suggests that protealysin may play a key role in *S. proteamaculans* invasion. This review addresses the mechanisms underlying protealysin’s involvement in bacterial invasion, highlighting the main findings as follows. Protealysin can be delivered into the eukaryotic cell by the type VI secretion system and/or by bacterial outer membrane vesicles. By cleaving actin in the host cell, protealysin can mediate the reversible actin rearrangements required for bacterial invasion. However, inactivation of the protealysin gene leads to an increase, rather than decrease, in the intensity of *S. proteamaculans* invasion. This indicates the presence of virulence factors among bacterial protealysin substrates. Indeed, protealysin cleaves the virulence factors, including the bacterial surface protein OmpX. OmpX increases the expression of the EGFR and β1 integrin, which are involved in *S. proteamaculans* invasion. It has been shown that an increase in the invasion of genetically modified *S. proteamaculans* may be the result of the accumulation of full-length OmpX on the bacterial surface, which is not cleaved by protealysin. Thus, the intensity of the *S. proteamaculans* invasion is determined by the balance between the active protealysin and its substrate OmpX.

## 1. Introduction

Penetration into eukaryotic cells provides a nutrient-rich growth environment as well as the ability to avoid contact with extracellular defenses against infection [1]. To protect themselves, eukaryotes have adapted intracellular processes to combat pathogens [2,3], while bacteria have developed many ways to circumvent host cell defenses [4,5]. Among the many virulence factors that mediate the penetration of bacteria into eukaryotic cells, bacterial proteases form a separate group of virulence factors. The serine protease HtrA is the most widespread and characterized as a regulator of bacterial virulence [6]. HtrAs are able to cleave cell-to-cell junction factors such as E-cadherin, and extracellular matrix proteins such as fibronectin, disrupting the epithelial barrier and facilitating bacterial access to host cells [6]. In addition, these proteases have been shown to be involved in bacterial invasion of eukaryotic cells [7]. One of the likely mechanisms for resolving bacterial invasion by protease is the regulation of the biogenesis of virulence factors SpeB and the hemolysin streptolysin S [8].

Bacterial metalloproteases participate in regulated intramembrane proteolysis and control cell envelope virulence factors of *Mycobacterium tuberculosis* [9]. Some secreted bacterial proteases become virulence factors by activating or inhibiting host cell matrix metalloproteases [10]. The substrates of metalloproteases can be the cell matrix (collagen, fibronectin and laminin) and cytoskeletal proteins (actin and tubulin) [11]. Studying the role of bacterial proteases is essential for understanding the mechanisms of bacterial invasiveness.

The facultative pathogen *Serratia proteamaculans* is capable of penetrating eukaryotic cells, and this ability was correlated with the appearance of the active metalloprotease protealysin in extracts of these bacteria [12]. Moreover, non-invasive *E. coli*, after transformation with a plasmid carrying the protealysin gene, acquired the ability to penetrate eukaryotic cells and were detected in the cytoplasm of infected cells using confocal and electron microscopy [13]. Based on these data, it was hypothesized that protealysin may play a key role in *S. proteamaculans* invasion. Further work summarized below was aimed at better understanding the mechanisms of protealysin involvement in bacterial invasion

## 2. Protealysin Can Penetrate Host Cells

**Figure 1:**
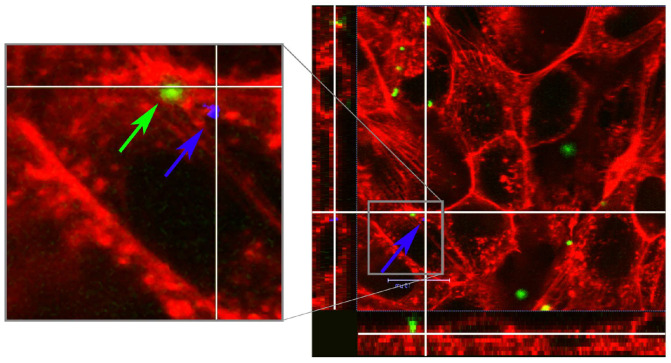
Localization of protealysin after incubation of Balb 3T3-SV40 fibroblasts with *S. proteamaculans*. Cytoskeleton was stained with rhodamine-phalloidin (red); bacteria were stained with FITC (green) and marked with green arrow; protealysin (marked with blue arrow) was stained with polyclonal rabbit anti-protealysin serum and the secondary Alexa647-conjugated antibody to rabbit IgG. The views on the left and below are projections of cells along the white lines. A higher magnification of the area enclosed by the gray frame is shown on the left. Modified from [14].

During host cells incubation with bacteria, protealysin penetrates into the eukaryotic cell (Figure 1) [14]. To deliver protealysin to the eukaryotic cell, bacteria can use the secretion system and/or bacterial outer membrane vesicles. *S. proteamaculans* can form outer membrane vesicles that carry the active protease protealysin [15]. On the other hand, invading bacteria use pore-forming type III, IV or VI secretion systems to deliver virulence factors into the host cell [16]. Serratia bacteria have a type VI secretion system (T6SS) [17]. This bacterial secretion system is used both to deliver proteins to neighboring bacteria and to deliver effector molecules to the host cell [18]. Many bacteria carry genes for several T6SSs, and, depending on conditions, synthesize T6SS specific for bacteria or for eukaryotic cells [19]. One of the components of the T6SS system is the effector protein VgrG, which, when penetrating into eukaryotic cells, remains associated with the secretion system built into the host cell membrane [20]. At the same time, the protein VgrG serves as a primer for the actin and microtubules polymerization [19,21]. The S. prot genome contains the *vgrG* gene and other genes for the proteins of the T6SS. Thus, the target of protealysin action may be located inside the host cell.

## 3. Regulation of the Dynamics of Actin Rearrangements by Protealysin

Cytoskeletal rearrangements in the host cell are necessary for bacterial penetration into eukaryotic cells [22]. Penetration of bacteria into eukaryotic cells involves the disassembly of actin structures at the site of bacterial–cell contact followed by actin polymerization in cell surface protrusions, which rise around the bacteria and allow its engulfment [22]. Bacterial effectors can mediate cytoskeletal rearrangements by regulating the activity of small GTPases and actin-binding proteins, or by directly interacting with actin. Many bacterial effectors mimic the natural activators of the small GTPases Rho, Rac and Cdc42 or stimulate host signaling pathways by functional mimicry of host GTPases, resulting in actin polymerization [23,24]. Other bacterial effectors stimulate bacterial uptake into host cells by forming complexes with actin binding proteins [25] or by inducing actin binding protein phosphorylation/dephosphorylation [26,27,28,29]. In addition, many toxins and bacterial factors interact directly with actin. Toxin RTX of *Vibrio cholerae* covalently cross-links actin monomers, thereby preventing the formation of functional filaments [30]. Toxin C2 of *Clostridium botulinum* and ADP-ribosyltransferase SpvB of *Salmonella enterica* induce ADP-ribosylation of actin at Arg177, thereby inhibiting actin polymerization [31,32]. On the other hand, the bacterial protein SipA of *Salmonella typhimurium* inhibits the depolymerization of actin filaments by mechanically stabilizing the filaments by binding actin subunits in opposing filaments [33,34]. Thus, bacterial factors entering the host cell can regulate actin dynamics, providing cytoskeletal rearrangements necessary for bacterial invasion.

**Figure 2:**
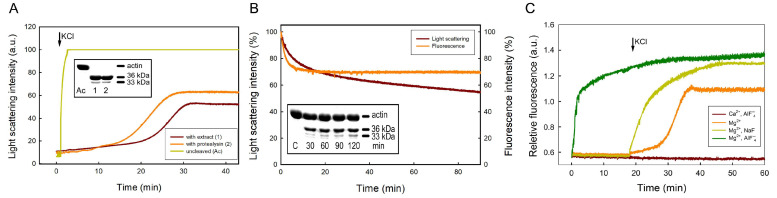
(**A**) Polymerization of the protealysin cleaved G-actins. Ca-G-actin was cleaved with protealysin of bacterial extracts of recombinant *E. coli* expressing the protealysin (1) and purified protealysin (2). Ac, nondigested actin (insert). Cleaved Ca-G-actin was transformed into Mg-G-actin and polymerized with 0.1 M KCl (arrow). (**B**) Effects of proteolysis on F-actin polymerization status. At time zero, the lysate of recombinant *E. coli* expressing the protealysin was added to Mg-F-actin. F-actin cleavage was monitored by SDS/PAGE (insert). Control, nondigested actin. (**C**) Effects of aluminum and sodium fluoride on polymerization of the protealysin cleaved actin. Protealysin-cleaved Ca-G-actin was transformed into Mg-G-actin. At time zero, Ca- and Mg-actins were either complemented with 1 mM AlF_4_^−^ (Ca^2+^, AlF_4_^−^ and Mg^2+^, AlF_4_^−^, respectively) followed by the addition of 0.1 M KCl (arrow) or directly polymerized with 0.1 M KCl in the absence (Mg^2+^) or presence of 5 mM NaF (Mg^2+^, NaF). All panels are modified from [14,37].

Purified protealysin and extracts of *S. proteamaculans* hydrolyze the Gly42–Val43 peptide bond in globular actin within the DNase I-binding loop [12] (Figure 2A insert). When the amount of purified enzyme is increased to a ratio with actin of 1:50, the 36 kDa fragment of actin formed as a result of hydrolysis of the Gly42–Val43 bond undergoes further proteolysis in the Gly63–Ile64 and Thr66–Ile67 bonds in the nucleotide cleft of the molecule with the formation of a 33 kDa fragment [12]. Actin cleaved at the Gly42 and Val43 bond retains its native conformation [35,36], but such actin completely loses the ability to polymerize in the presence of Ca^2+^ [14,36] (Figure 2A). The ability of cleaved actin to polymerize is partially restored when tightly bound Ca^2+^ is replaced with Mg^2+^ [14,36] (Figure 2A). However, actin cleaved by protealysin has a lower degree of polymerization and a 30-fold higher rate of exchange of subunits in the polymer compared to intact actin [14] (Figure 2A). Thus, cleavage of the peptide bond between Gly-42 and Val-43 in globular actin causes local conformational changes that weaken intermonomer contacts during actin polymerization and lead to increased polymer dynamics.

In a eukaryotic cell, globular actin is associated with actin-binding proteins or forms filaments (F-actin) and is not accessible to proteolysis by most proteases. The lysate of bacteria synthesizing protealysin cleaves up to 40% of F-actin [14] (Figure 2B). Protealysin cleaves the bond between Gly-42 and Val-43 in filamentous actin subunits, causing filament instability similar to the instability of filament polymerized from protealysin-cleaved globular actin [14] (Figure 2B, insert). Increased filament dynamics from protease-cleaved monomers between Gly-42 and Val-43 can be reversed by phalloidin [36], aluminum and sodium fluorides [14,37] (Figure 2C), as well as the actin-binding proteins gelsolin [38], myosin subfragment 1 [39] and tropomyosin [40]. Thus, modifications of the actin cytoskeleton produced by protealysin can be restored by actin-binding proteins and can be used by bacteria to rearrange actin filaments necessary for their own penetration into eukaryotic cells.

## 4. *S. proteamaculans* Invasion without Protealysin

**Figure 3:**
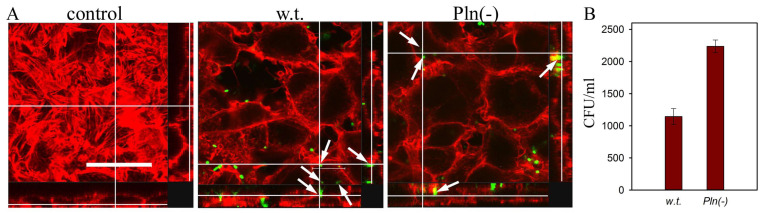
Invasion of M-HeLa cells by *S. proteamaculans* (w.t.) and *S. proteamaculans* Pln(-) containing the inactivated protealysin gene. (**A**) Confocal microscopy images of M-HeLa cells incubated with bacteria. Cytoskeleton was stained with rhodamine phalloidin (red); bacteria (arrows) were stained with FITC (green). The views on the right and bottom are projections of cells along the white lines. Bar, 30 μm. (**B**) Quantitative evaluation of the susceptibility of M-HeLa cells to invasion. Modified from [41].

Based on previous data, protealysin was hypothesized to be a key regulator of *S. proteamaculans* invasion. However, a quantitative assessment of the intensity of invasion of bacteria *S. proteamaculans* showed that inactivation of the protealysin gene did not lead to a decrease, but to a two-fold increase in the intensity of bacterial invasion [41] (Figure 3A,B). This indicates that, in addition to protealysin, additional virulence factors are also involved in the invasion of *S. proteamaculans*. In addition to actin-specific proteases, *Serratia* virulence factors include the pore-forming toxin (hemolysin) ShlA, [42,43], extracellular protease [44], which promotes the invasion of *Serratia marcescens*. The pore-forming hemolysin ShlA induces *S. marcescens* invasion of epithelial cells by enhancing host cell vacuolation and lysis [42]. And the intensity of invasion of the *S. marcescens* strain, defective in hemolysin synthesis, into epithelial cells RT112 is almost 90 times lower compared to the intensity of wild strain *S. marcescens* invasion [42]. Along with hemolysin, *S. marcescens* secretes the extracellular protease serralysin, which is cytotoxic to mammalian cells [44]. Serralysin synthesis in both *S. marcescens* and genetically engineered *E. coli* correlates with bacterial cytotoxicity [44]. In addition, serralysin, by cleaving transmembrane receptors, is capable of thereby modulating host inflammatory and immune responses [45]. It was found that not only *S. marcescens*, but also *S. proteamaculans* synthesize the active pore-forming toxin ShlA and serralysin [46]. Thus, in addition to protealysin, serralysin and the pore-forming toxin may be involved in the penetration of *S. proteamaculans* into eukaryotic cells.

**Figure 4:**
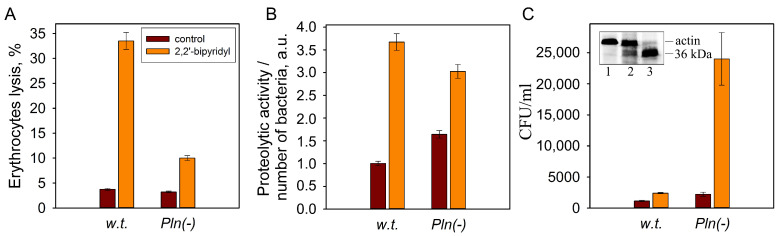
Effects of iron-depletion on the activity of the bacterial virulence factors of *S. proteamaculans* (w.t.) and *S. proteamaculans* Pln(-) with an inactivated protealysin gene. The effect of 2,2′-bipyridyl in the growth medium on the hemolytic activity characterizing activity of ShlA toxin (**A**), the activity of extracellular metalloprotease serralysin (**B**) and the intensity of invasion (**C**). The insert shows the actin-hydrolyzing activity in the extracts of *S. proteamaculans* (w.t.) grown in the absence (lane 2) or presence (lane 3) of 2,2′-bipyridyl. Control actin (lane 1). Modified from [41].

Since the iron content in the body’s environment during infections is limited due to sequestration by host proteins such as lactoferrin, transferrin and hemoglobin, a decrease in iron concentration in the environment is one of the factors signaling the entry of bacteria into the body. The iron concentration in the environment can regulate the expression of virulence genes. Expression of virulence factors of *Pseudomonas syringae* [47] and *Staphylococcus aureus* [48] is induced under conditions of iron deficiency in the environment. A decrease in iron concentration in the environment leads to the activation of pore-forming toxins of pathogenic bacteria including *Listeria monocytogenes* [49], *Yersinia ruckeri* [50], and *S. marcescens* [51]. When *S. proteamaculans* grows in an iron-depleted environment, the activity of both hemolysin and serralysin increases [46] (Figure 4A,B). This increase in the activity of virulence factors leads to a three-fold increase in the intensity of *S. proteamaculans* invasion [41] (Figure 4C). However, quantitative analysis showed that growth in an iron-depleted environment leads to a 10-fold increase in the intensity of invasion of *S. proteamaculans* with an inactivated protealysin gene [41] (Figure 4C). This indicates that among the virulence factors of *S. proteamaculans* there are protealysin substrates, and their expression increases during growth in an iron-depleted environment.

## 5. Bacterial Substrates of Protealysin

Protealysin can cleave RecA protein in vitro. RecA plays a role in regulating bacterial adhesion and invasion. The RecA-dependent pathway mediates the binding of *Staphylococcus aureus* to the extracellular matrix protein fibronectin [52] and regulates the type III secretion system of enteropathogenic *E. coli* [53]. In addition, RecA has been shown to be activated during adhesion and invasion of Caco-2 intestinal epithelial cells by *Listeria monocytogenes*, and deletion in the RecA reading frame in these bacteria reduces the adhesion and invasion of Caco-2 cells [54]. Thus, cleavage of RecA may reduce bacterial virulence. Apparently, the accumulation of uncleaved RecA in *S. proteamaculans* bacteria with inactivated protealysin may be the cause of increased bacterial invasion.

Using mass spectrometry, an analysis was carried out of proteins degraded by protealysin in extracts and the membrane fraction of *S. proteamaculans* at the early stationary phase of bacterial growth, when protealysin was not yet active [12]. Outer membrane protein OmpX, DNA starvation/stationary phase protection protein Dps, uridine phosphorylase, peroxiredoxin C, glyceraldehyde-3-phosphate dehydrogenase, molecular chaperone OsmY, outer membrane protein OmpW, and thiol-disulfide interchange protein DsbA were found as potential protealysin substrates in the membrane fraction [41]. The molecular chaperone GroEL, galactose/glucose ABC transporter substrate-binding protein MglB, triol-disulfide isomerase DsbA, molecular chaperone OsmY and amino acid ABC transporter substrate-binding protein were identified as protealysin substrates in the bacterial extract [41]. Almost all of the identified protealysin substrates can mediate bacterial invasion. Molecular chaperone GroEL, outer membrane protein W, galactose/glucose ABC transporter substrate-binding protein MglB, glyceraldehyde- 3-phosphate dehydrogenase A, amino acid ABC transporter substrate-binding protein are involved in adhesion to the host cell surface [55,56,57,58,59,60,61,62,63,64]. Thiol-disulfide isomerase DsbA is a component of the bacterial type VI secretion system, specific to the transport of bacterial virulence factors into eukaryotic cells [65,66,67]. DNA starvation/stationary phase protection protein Dps, peroxiredoxin C, and molecular chaperone OsmY themselves are virulence factors [68,69,70]. In addition, it has been shown that inactivation of the OmpX can lead to a decrease in bacterial invasion, as a result of a decrease in adhesion by pathogenic *Escherichia coli* (ExPEC) and *Yersinia pestis* or without affecting the adhesion of *Salmonella enterica* and *Cronobacter sakazakii* [71,72,73,74,75].

Protealysin cleaves OmpX in an unlimited manner in bacterial lysate [76]. However, analysis of the structure of OmpX [77] showed that most of the sites specific for cleavage by protealysin are located in membrane-integrated β-layers and are not accessible for protealysin. Sites available for cleavage by protealysin are located in the N- and C-terminal loops on the bacterial surface [77]. Based on the substrate specificity of protealysin [12,78], it was proposed that full-length OmpX was cleaved between Gly40 and Val41. In this case, the 23 N-terminal amino acids form a signal propeptide, the cleavage of which is necessary for the activation of OmpX [79]. Thus, protealysin may be a regulator of the activity of the bacterial surface protein OmpX.

## 6. Regulation of OmpX by Protealysin in Bacteria

**Figure 5:**
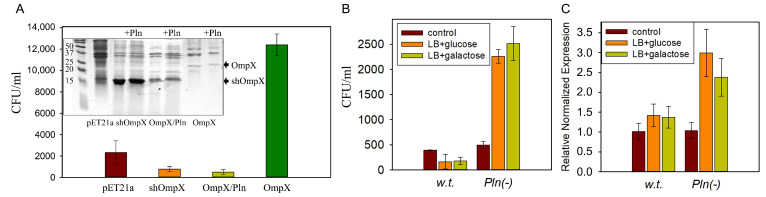
(**A**) Effects of recombinant truncated OmpX accumulation in *E. coli* on the adhesion to the host cell surface. Quantitative evaluation of the adhesion to M-HeLa cells of bacteria *E. coli* (pET21a), *E. coli* (shOmpX), synthesizing truncated OmpX, *E. coli* (OmpX/Pln), synthesizing both full-length OmpX and protealysin, *E. coli* (OmpX), synthesizing full-length OmpX. The insert shows the membrane fractions of *E. coli* bacteria containing recombinant truncated and full-length OmpX, before and after incubation with purified protealysin. (**B**) Invasion of 3T3-SV40 cells by *S. proteamaculans* (w.t.) and *S. proteamaculans* Pln(-) grown in LB medium in the absence and presence of glucose or galactose. (**C**) Expression of OmpX in bacteria grown in LB medium in the absence and presence of glucose or galactose. All panels are modified from [41].

Using *E. coli* that synthesizes a truncated OmpX without the 40 N-terminal amino acids, and *E. coli* that synthesizes both OmpX and protealysin, it was shown that a truncated OmpX is produced in both bacteria. Truncated OmpX can integrate into the bacterial membrane, after which it becomes inaccessible for further cleavage by protealysin [41] (Figure 5A, insert). Transformation with a plasmid carrying the full-length OmpX gene increases *E. coli* adhesion to the host cell surface by three times, but it does not affect the ability to penetrate eukaryotic cells [41,76] (Figure 5A). However, transformation with a plasmid carrying a truncated OmpX gene without 40 N-terminal amino acids or both the OmpX and protealysin genes does not enhance *E. coli* adhesion [41]. Thus, the intensity of *S. proteamaculans* invasion as a result of inactivation of the protealysin gene may result from the accumulation of full-length OmpX on the bacterial surface. In addition, in the presence of glucose or galactose, the invasive activity of *S. proteamaculans* decreased two-fold, while the invasive activity of *S. proteamaculans* with an inactivated protealysin gene increased four-fold (Figure 5B) [41]. These results correlated with an increase in the OmpX expression in response to added sugars only in *S. proteamaculans* with an inactivated protealysin gene (Figure 5C) [41].

As described above, in the presence of the iron chelator 2,2′-bipyridyl, the hemolytic toxin ShlA and serralysin are activated, which leads to an increase in the intensity of *S. proteamaculans* invasion. However, 2,2′-bipyridyl activates serralysin and the ShlA toxin 2–3 times more strongly in the wild strain bacteria than in bacteria with an inactivated protealysin gene; and on the contrary, the increase in intensity of invasion is reduced about five-fold [41] (Figure 4). Depletion of iron reserves leads to the accumulation of OmpX in bacteria [80]. In addition, 2,2′-bipyridyl enhances protealysin activity, which may lead to the accumulation of truncated instead of full-length OmpX and contributes to the attenuation of the increase in the intensity of invasion of the *S. proteamaculans* [41]. Thus, the greatly enhanced invasion of *S. proteamaculans* with an inactivated protealysin gene upon iron depletion may be a consequence of the accumulation of full-length OmpX in the absence of protealysin activity.

**Figure 6:**
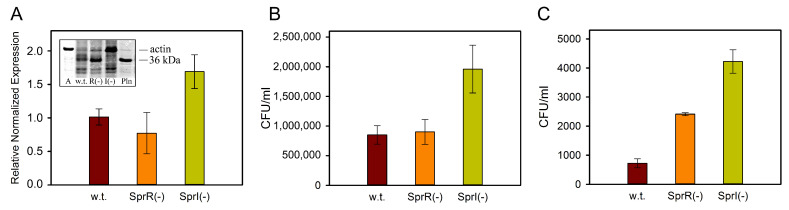
Effect of the Quorum Sensing system inactivation. (**A**) Expression of OmpX in *S. proteamaculans* with inactivated QS system. S12 ribosomal protein served as an internal control. The insert shows the actin-hydrolyzing activity in the extracts of *S. proteamaculans* and purified protealysin. A—control actin. Quantitative evaluation of the susceptibility of M-HeLa cells to adhesion (**B**) and invasion (**C**) by *S. proteamaculans* (w.t.), *S. proteamaculans* SprR(-) containing the inactivated *sprR* receptor gene. and *S. proteamaculans* SprI(-) containing the inactivated AHL synthase *sprI* gene. Modified from [84,85].

*S. proteamaculans* synthesize active protealysin during the stationary growth phase, when the bacterial population density is maximal [12]. In response to changes in population density, gene expression, including virulence factors, is regulated by the Quorum Sensing system (QS). *S. proteamaculans* have a LuxI/LuxR type QS system consisting of the AHL synthase SprI and the regulatory receptor protein SprR [81]. In the classical model of the QS system, binding to AHL stabilizes LuxR-type proteins, allowing them to bind DNA and activate transcription of target genes, including the AHL synthase gene [82]. However, the *S. proteamaculans* QS system differs from the classical QS system in that the *sprI* AHL synthase gene and the *sprR* receptor protein gene are convergently transcribed and overlap in their terminal regions [81], making AHL synthase independent of the SprR protein [83]. Accordingly, only a mutation in the AHL synthase *sprI* gene, but not a mutation in the *sprR* receptor gene, led to a decrease in exoprotease and chitinolytic activity and the ability of *S. proteamaculans* to form biofilms [83]. Likewise, the activity of protealysin is affected only by inactivation of the *sprI* AHL synthase gene, but not by inactivation of the *sprR* receptor gene [84,85] (Figure 6A, insert). Inactivation of the *sprI* AHL synthase gene led to a decrease in protealysin activity and an increase in the expression of the surface protein OmpX, which led to increased adhesion of these bacteria, but not bacteria with an inactivated *sprR* receptor gene [84,85] (Figure 6A,B). Increased bacterial adhesion led to an increase in the intensity of *S. proteamaculans* invasion as a result of inactivation of the *sprI* AHL synthase gene [84] (Figure 6C). These results suggest that synthesis of AHLs controls the invasion of *S. proteamaculans* by accumulation on the bacterial surface of full-length OmpX not cleaved by protealysin [84].

## 7. Interaction of OmpX with Host Cell Receptors

**Figure 7:**
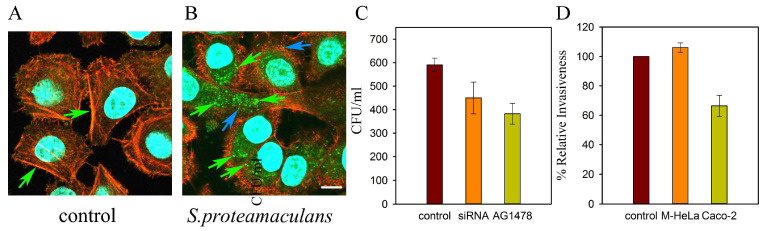
Distribution of EGFR in M-HeLa cells before (**A**) and after (**B**) incubation with *S. proteamaculans.* Cytoskeleton was stained with rhodamine-phalloidin (red); EGFR (green) was stained with antibodies and the secondary Alexa488-conjugated antibody to rabbit IgG; and DNA (blue turquoise) was stained with DAPI. Bacteria (blue turquoise) are indicated with a blue arrow based on the blue channel image. EGFR is indicated with a green arrow. Scale bar: 10 µm. Modified from [87]. (**C**) Effect of treating M-HeLa cells with siRNA targeting EGFR and inhibitor of EGFR phosphorylation (tyrphostin AG 1478) on cell sensitivity to *S. proteamaculans* invasion. (**D**) Effect of transfection of M-HeLa and Caco-2 cells with siRNA targeting E-cadherin on cell sensitivity to *S. proteamaculans* invasion. Control—intensity of invasion into cells transfected with siRNA containing scrambled nucleotide sequence. The number of intracellular bacteria was estimated as a percentage, taking the number of intracellular bacteria in control samples as 100%. Modified from [89].

OmpX family proteins have shown the ability to bind to the epidermal growth factor receptor (EGFR) [86]. Using siRNA targeting EGFR, this receptor was shown to be involved in *S. proteamaculans* invasion [87] (Figure 7C). In control cells, the EGFR is localized with actin along the cell perimeter (Figure 7A, indicated by arrows). Infection with bacteria leads to the formation of EGFR-carrying endosomes [87,88] (Figure 7A,B). Only single endosomes are localized with the EGFR and bacteria (Figure 7B, indicated by blue arrows). The remaining EGFR-carrying endosomes do not transport bacteria (Figure 7B, all other endosomes, including indicated by green arrows). An inhibitor of EGFR phosphorylation reduced the sensitivity of host cells to invasion by *S. proteamaculans*, indicating the involvement of EGFR signal transduction in the invasion of these bacteria [87] (Figure 7C). Thus, contact of *S. proteamaculans* with a eukaryotic cell triggers a signaling mechanism in which the EGF receptor transmits a signal from the surface of the host cell.

EGFR can interact with E-cadherin on the surface of eukaryotic cells [90]. E-cadherin accumulates in M-HeLa cells in a shortened form and only in response to incubation with the *S. proteamaculans* [89]. Bacteria can promote invasion by cleaving E-cadherin to produce the 80-kDa soluble E-cadherin fragment (sE-cad) [91] by bacterial proteases or by recruiting host proteases such as ADAM sheddases and matrix metalloproteinases (MMP) [92]. Shidase ADAM10 is activated by Ca^2+^ influx into host cells through the formation of pores in the cytoplasmic membrane [93] by the ShlA toxin of *S. marcescens* among others [94]. However, accumulation of Ca^2+^ in M-HeLa cells requires more than 2 h of incubation with *S. proteamaculans*, and the GI 254023X inhibitor of ADAM10 does not affect the invasion of these bacteria [89]. The matrix metalloproteinase MMP-2 of eukaryotic cells can also cleave E-cadherin to form sE-cad. According to zymography data, protealysin converts the precursor of the matrix metalloproteinase MMP-2 into a polypeptide with a molecular weight of 66 kDa, characteristic of mature MMP-2, which indicates the possibility of activation of MMP-2 by protealysin [12]. The sE-cad can associate with intact E-cadherin present on other cells to alter cadherin-dependent cellular behavior [95]. In addition, sE-cad can bind to EGFR and stimulate EGFR phosphorylation, playing a role in EGFR signaling independent of traditional EGFR ligands [95,96]. On the other hand, according to electron microscopy data, more than 99% of protealysin accumulates in the form of an inactive precursor and is associated with the bacterial cell [97]. The enzyme matures only after bacterial lysis, which can occur during incubation with a eukaryotic cell. This suggests that *S. proteamaculans* protealysin is released from bacteria in response to an external stimulus, including contact with a eukaryotic cell. However, addition of protealysin to the medium did not lead to the penetration of non-invasive *E. coli* into eukaryotic cells [13]. In addition, quantitative assessment of the intensity of invasion showed that the appearance of protealysin in the growth medium does not affect the invasion of *S. proteamaculans* [41]. Using siRNA targeting E-cadherin, it was shown that this receptor is involved in the invasion of *S. proteamaculans* only into Caco-2 cells, in which full-length E-cadherin remains after incubation with bacteria [89] (Figure 7D). Thus, E-cadherin can participate in *S. proteamaculans* invasion only in its uncleaved form. However, the entry of these bacteria into M-HeLa cells indicates the presence of additional EGFR partners that are involved in bacterial invasion.

**Figure 8:**
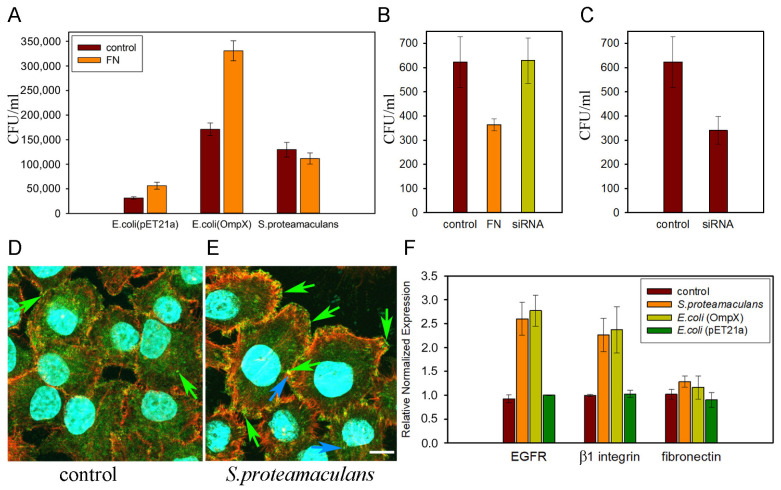
(**A**) Adhesion of *E. coli* (pET21a; control), *E. coli* (OmpX) synthesizing OmpX and *S. proteamaculans* to fibronectin. (**B**) The effect of treating of *S. proteamaculans* with fibronectin and M-HeLa cells with siRNA targeting fibronectin on the intensity of bacterial invasion. Control, bacterial invasion of cells without treatment. (**C**) Effect of treating M-HeLa cells with siRNA targeting β1 integrin on cell sensitivity to *S. proteamaculans* invasion. Control—M-HeLa cells transfected with siRNA containing scrambled nucleotide sequence. (**D**,**E**) Distribution of β1 integrin in M-HeLa cells before (**D**) and after (**E**) incubation with *S. proteamaculans.* Cytoskeleton was stained with rhodamine-phalloidin (red); β1 integrin (green) was stained with antibodies and the secondary Alexa488-conjugated antibody to mouse IgG; and DNA (blue turquoise) was stained with DAPI. Bacteria (blue turquoise) are indicated with a blue arrow based on the blue channel image. β1 integrin is indicated with a green arrow. Scale bar: 10 µm. (**F**) Effect of bacterial infection on expression levels of EGFR, β1 integrin and fibronectin in the host cell. Control—uninfected M-HeLa cells. All panels are modified from [87].

In addition to the EGFR, OmpX can interact with extracellular matrix protein fibronectin [72]. The addition of fibronectin increases the adhesion of *E. coli* synthesizing OmpX, but fibronectin in the medium does not affect the adhesion of *S. proteamaculans* and even reduces their invasion [87] (Figure 8A,B). Using siRNA targeting fibronectin, it was confirmed that fibronectin is not involved in *S. proteamaculans* invasion [87] (Figure 8B). On the other hand, fibronectin binds to α5 and β1 integrins heterodimer on eukaryotic cells surface [98], and using siRNA targeting β1 integrin, the involvement of this receptor in the *S. proteamaculans* invasion has been shown [87] (Figure 8C). Moreover, bacterial infection leads to the accumulation of α5 and β1 integrins along the cell perimeter [87,88] (Figure 8D,E). Integrins can use the EGFR as a transmitter in signaling pathways downstream of the cell matrix [99]. The interaction between EGF receptor and β1 integrin is disrupted by the MβCD [100]. Disruption of rafts by the MβCD reduces the sensitivity of cells to the *S. proteamaculans* [101], suggesting the possibility that the bacterium binds to β1 integrin and triggers invasion-promoting signaling mechanisms through EGFR.

The presence of receptors on the surface of the host cell is highly dependent on the cell line [88], but the sensitivity of cells of different cell lines to *Serratia* is almost the same [84,85,102]. It turned out that the contact of OmpX with the surface of an eukaryotic cell leads to an increase in the expression of the integrin β1 and EGFR genes, which determine the intensity of *S. proteamaculans* invasion [87] (Figure 8F).

## 8. Concluding Remarks

**Figure 9:**
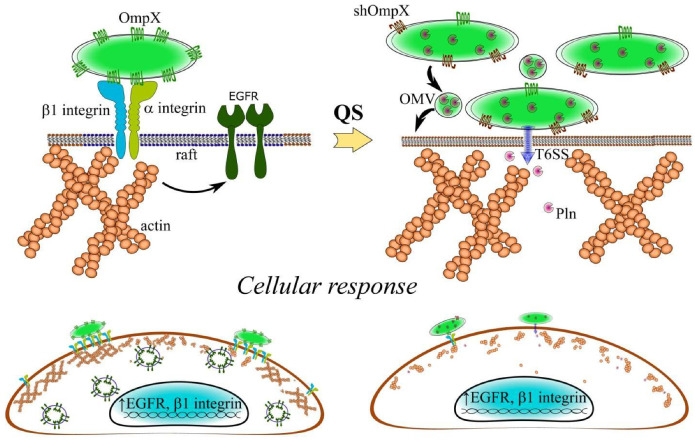
Scheme of interaction between *S. proteamaculans* and host cell. By contacting host cell receptors, the bacterial surface protein OmpX (**top left**) increases adhesion and causes increased expression of EGFR and β1 integrin (**bottom**). Infection with bacteria leads to the formation of EGFR-containing endosomes and accumulation of β1 integrin on the surface of human cells (**bottom left**). When the bacterial population density increases (arrow), the bacterial QS system activates the protease protealysin, which cleaves de novo-synthesized OmpX to form a truncated shOmpX (**top right**). The truncated shOmpX is not involved in bacterial adhesion. On the other hand, protealysin enters the host cell via the type VI secretion system (T6SS) and/or bacterial outer membrane vesicles (OMVs) (**top right**). By cleaving actin in the host cell, protealysin can mediate actin rearrangement necessary for bacterial invasion (**bottom right**).

By contacting host cell receptors, the bacterial surface protein OmpX increases adhesion and causes increased expression of EGFR and β1 integrin, which are involved in the invasion of *S. proteamaculans* (Figure 9). Infection with bacteria leads to the formation of EGFR-containing endosomes and accumulation of β1 integrin on the surface of human cells. During the late stationary stage of growth, the bacterial QS system activates the protease protealysin, which cleaves de novo-synthesized OmpX. The truncated OmpX is not involved in bacterial adhesion. However, the full-length OmpX synthesized earlier remains on the surface of the bacterium. On the other hand, protealysin can be delivered into the eukaryotic cell by the type VI secretion system and/or by bacterial outer membrane vesicles. By cleaving actin in host cells, protealysin can mediate the reversible disassembly of perimembrane actin required for bacterial invasion. Thus, the intensity of *S. proteamaculans* invasion is determined by the balance between the active protealysin and its substrate OmpX.
